# 3D Printed Sensors for Biomedical Applications: A Review

**DOI:** 10.3390/s19071706

**Published:** 2019-04-10

**Authors:** Tao Han, Sudip Kundu, Anindya Nag, Yongzhao Xu

**Affiliations:** 1 DGUT-CNAM Institute, Dongguan University of Technology, Dongguan 523106, China; hant@dgut.edu.cn (T.H.); xuyz@dgut.edu.cn (Y.X.); 2 CSIR-Central Mechanical Engineering Research Institute, Durgapur, West Bengal 713209, India; sudip.skundu.94@gmail.com

**Keywords:** 3D printed sensors, fused deposition modelling, stereolithography, selective laser sintering, inkjet, polyjet, digital light processing

## Abstract

This paper showcases a substantial review on some of the significant work done on 3D printing of sensors for biomedical applications. The importance of 3D printing techniques has bloomed in the sensing world due to their essential advantages of quick fabrication, easy accessibility, processing of varied materials and sustainability. Along with the introduction of the necessity and influence of 3D printing techniques for the fabrication of sensors for different healthcare applications, the paper explains the individual methodologies used to develop sensing prototypes. Six different 3D printing techniques have been explained in the manuscript, followed by drawing a comparison between them in terms of their advantages, disadvantages, materials being processed, resolution, repeatability, accuracy and applications. Finally, a conclusion of the paper is provided with some of the challenges of the current 3D printing techniques about the developed sensing prototypes, their corresponding remedial solutions and a market survey determining the expenditure on 3D printing for biomedical sensing prototypes.

## 1. Introduction

The use of sensors for ubiquitous monitoring purposes has happened for quite some time [1,2,3,4]. In every automated industry like robotics, aeronautics and aerospace, biomedical devices and the manufacturing industry, sensors are being used to detect changes of the environment and transfer data to a monitoring unit [5]. For example, in biomedical studies, wearable sensors are used to analyse the physiological parameters of human beings. In earlier days, when semiconducting sensors were popular, researchers used silicon-based sensors to a large extent [6,7] for monitoring different industrial [8,9] and environmental [10,11] applications. Even though they served a great purpose for non-healthcare applications, silicon sensors for biomedical sensing were pretty limited. Although currently a lot of micro and nano-sensors are being fabricated based on silicon substrates [12,13,14], certain disadvantages like temperature dependence, low signal, high noise and high cost are some of the demerits which limit their uses. Apart from this, the biggest disadvantage lies in their non-biocompatible nature, which makes the resulting sensors unsuitable for biomedical applications. Without a biocompatible nature, sensors developed for biomedical applications cannot be considered for ubiquitous or implantable applications. Another disadvantage of silicon sensors lies in their irregular behaviour at very low frequencies, which makes it compulsory to operate them on a wider bandwidth of frequencies. Sensor operation at high frequencies requires more input power, thus increasing the cost of the total sensing system. The advent of flexible sensors [15,16] has brought about a lot of changes in certain attributes of the fabricated prototypes, thus minimising some of the mentioned demerits of silicon-based sensors. For flexible sensors, a wide range of processing materials have been processed to fabricate prototypes for different kinds of applications. For the substrate part, different kinds of polymers like polydimethylsiloxane (PDMS) [17,18], polyethylene terephthalate (PET) [19,20], polyimide (PI) [21,22] have been used. Similarly, carbon nanotubes (CNTs) [23,24], graphene [25,26], and gold nanoparticles [27,28] are some of the common conductive materials that have been used to develop the electrode part of the sensing prototypes. The conjugation of these individual polymeric substrates [29,30,31] and electrodes [32,33,34] plays their role in deciding the resultant electrical, mechanical and thermal characteristics of the prototypes. Among the fabrication techniques that have been available for developing flexible sensors, some of the commonly used ones are photolithography [35,36,37], screen printing [38,39,40], laser cutting [16,41,42], contact printing [43,44,45] and 3D printing [46,47]. Among them, the 3D printing technique has become very popular for its distinct advantages over other mentioned techniques. Comparatively, 3D printing requires a lesser number of steps and manual labour to complete the prototypes in comparison to certain conventional techniques like photolithography that are used for the fabrication of the sensors. Once the prototype is designed and uploaded to the system, the sensor is fabricated accordingly without much human intervention. Secondly, the developed sensing prototypes can be customised in accordance with the application of the sensors. One of the 3D printing techniques named Digital Light Processing (DLP) has been used to develop sensors with active components which changes its shape accordingly. Thirdly, along with the simple fabricating principles for 3D printed sensors, the prototypes can be developed with high accuracy, repeatability and resolution [48]. The range of materials that can be processed using this method is also higher than the other lithography techniques. This makes it easier to fabricate a sensor with multifunctional attributes using 3D printing techniques. In comparison to a screen-printing method which is one of the conventional methods to develop flexible sensors, the 3D printed sensors have higher durability and material strength, thus increasing its robustness and capability to withstand operations in harsh conditions. Another advantage of this technique is the high reusability, without compromising on their efficiency and sensitivity of the sensors. Some of the basic advantages of 3D printing lies in its simple fabrication process, quick production, less manual labor, less waste generation and risk mitigation [49]. In comparison to other lithography techniques like photolithography and screen-printing have certain disadvantages associated with them. For example, some of the disadvantages related to photolithography are degradation of the quality of the exposure regions as a result of the transfer of particles, high possibility of misalignment of the designs during the exposure process, high probability of damage of the designs during the etching process. Some of the disadvantages related to the screen-printing process are not economical for unit production, limited color mixing and multistep process.

**Figure 1:**
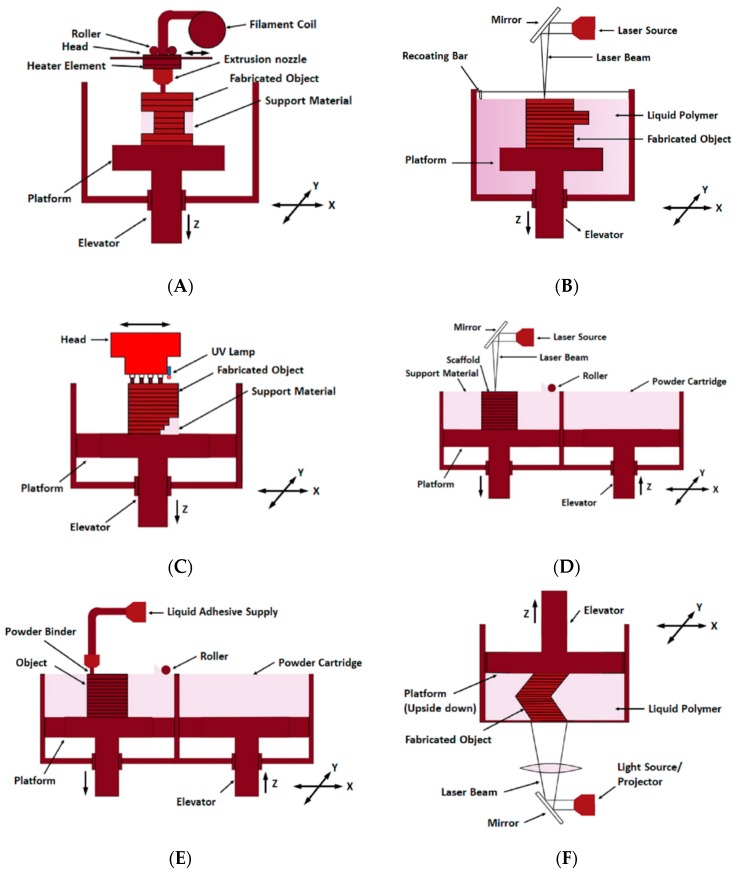
(**A**) Fused deposition modelling (**B**) Stereo-lithography (**C**) Polyjet Process (**D**) Selective laser sintering (**E**) 3D Inkjet printing (**F**) Digital light processing.

Although for the last forty years, the 3D printing technology has been developed and applied for manufacturing products including electronic parts [50], research and application of this technique in medical fields have been carried out since early 2000 by different biotech companies and research groups. One of the common techniques called the additive manufacturing (AM) technology [51,52] had been popularised over the years in the 3D printing technique to form the prototypes [53,54]. Certain techniques including powder bed fusion process, photopolymerisation, lamination, binder jetting and material extrusion fall under this process. Among all of these, the inkjet printing technique creates 3D prototypes with the highest flexibility [55]. The versatility of this process is very high as different kinds of materials like metals, ceramics, plastics and even the living cells can be deposited onto the substrates from the prototypes [52,56]. Due to the easy operating principle and quick fabrication of complex 3D models, the additive manufacturing technique has a wide spectrum of applications [57,58,59,60,61]. Different kinds of physiological parameters including blood pressure, heart rate, body motion, respiration rate, brain activity and skin temperature, have been measured with the 3D printed sensing components being integrated with the biomedical devices [62,63,64]. Generally, the 3D printed sensors are fabricated either by integrating the sensor in the printed platform or by direct printing of sensing component [65]. Fabricating 3D intricate designed compounds and nanosensors via 3D printing technology offers advantage and potential in terms of high efficiency and sensitivity [66,67]. Also, fabricating a sensor or a sensing platform being embedded to a sensor requires less manual setting for the additive manufacturing process than other traditional manufacturing methods. Hence, even though, the mass production of 3D printed products is not acceptable in manufacturing industries, it is mostly acceptable where rapid modifications are required in the sensor manufacturing industries [68]. This is the reason for the need of this cutting-edge technology to meet the different requirements for the functional elements of a sensor. Figure 1 shows schematic diagrams of the fabrication processes of the six different types of 3D printing that are available in the current day. 

Each of the technologies, namely fused deposition modelling (FDM), stereolithography (SLA), polyjet process, selective laser sintering (SLS), 3D inkjet printing and DLP, differs from the other ones with respect to different parameters including fabricated prototype, required time to develop each prototype, ability to process different raw materials, repeatability, resolution, accuracy and sensors developed for different applications. The explanation of each of these six types has been done in the succeeding sections where some of the significant research works related to the fabrication of sensors for biomedical applications for each of these types have been highlighted.

Table 1 gives a comparative study of the described 3D printing methods in terms of their principles, materials, resolution and applications. It is seen from the table that each of these fabrication techniques has their associated advantages, which are exploited for different kinds of biomedical-related applications. Among these six types, the most common type is the FDM one, which has been largely used to develop prototypes for electrochemical sensing purposes. Others like FDM, SLA and ink-jet printing have also been considered for forming prototypes since they can be developed with lower resolutions. Polyjet and SLS processes are mostly used for forming sensors which are employed for cell culture applications. Table 2 shows another comparative study of certain parameters showcasing the advantages and disadvantages associated with each technique. It is seen that even though all these techniques are useful to a certain range, only inkjet printing and DLP are capable provide prototypes with the highest repeatability. The poly jet process has largely used nowadays for 4D printing to print prototypes having active components in them.

Although a lot of review papers have been written in recent times describing the use of 3D printing technology for sensing purposes [48,87,88], none of them has specifically talked about the development of 3D printed sensors for biomedical applications. This review paper highlights some of the significant works done on the fabrication of 3D printed sensors for biomedical applications. The review article has been divided into four different sections which start with a brief introduction on the importance of sensors and 3D printing sensing in biomedical applications. Section 2 classifies the research works in six types based on their fabrication procedures. The challenges related to the current 3D printed biomedical sensors and some of the corresponding remedial solutions are explained in Section 3. The conclusion is given in the final section of the paper.

## 2. Types of 3D Printing for Biomedical Application

### 2.1. Fused Deposition Modelling 

Fused deposition modelling is a popular additive manufacturing technique for making 3D printed sensors for biomedical applications [89,90,91,92]. In this process, to make the 3D structure, a thin filament of thermoplastic polymeric material is fed and extruded in hot mushy condition from the heated nozzle of FDM machine for subsequent deposition onto a substrate. Thus, the thermoplastic polymeric material fabricates the cross sections of the 3D structure one after another. Before deposition of the subsequent layer, the previous layer cools down below its thermoplastic temperature. Acrylonitrile butadiene styrene (ABS), polylactic acid (PLA), wax blend, nylon, etc. are used as a printing material. 3D scaffolds seeded with living cells can be successfully printed using FDM without losing cell viability [93]. If the printing material is not properly tuned, the fabricated product shows lack of shape integrity and leakage. Surface finishing is often required to get the final product.

Krejcova et al. [94] used polylactide to fabricate a microfluidic chip by the extrusion process of a continuous molten filament at temperature 210 ℃. The melting head on a heated surface at a temperature of 40 ℃ consisted of three glassy carbon microelectrodes, each one having a diameter of 2.10 mm. This 3D printed chip has effective usage for electrochemical analysis of influenza virus using CdS quantum dots. Dias et al. [95] used a 3D printed batch injection analysis cell coupled with electrochemical detection and fabricated paper-based enzymatic reactors to detect the presence of glucose in artificial serum sample within a very short time. Using the 3D printer, the process exhibits the advantages of robustness and versatility with high analytical performance.

Leigh et al. [96] developed a simple conductive thermoplastic composite to print the electronic sensor which can sense the capacitance change and mechanical flexing. Kadimisetty et al. [97] developed a gravity flow microfluidic immunosensor from PLA using FDM to detect the three cancer biomarker proteins in serum simultaneously within 35 min. In this type of sensors, light-rechargeable supercapacitors were being used to supply the power. Henceforth, these sensors are appropriate for low and moderate resource medical surroundings. Su et al. [98] constructed a 3D-printed highly sensitive reactor via FDM for online monitoring of glucose and lactate in biological samples. This enzyme-based biosensor monitors specific brain biomolecules and investigates how the extracellular glucose and lactate present in the brain are complicatedly influenced in living animals with its high specificity, convenient fitting and reusability. Walzik et al. [99] modelled a smaller and lighter device using AutoCAD software and manufactured the 3D printed parts from ABS polymer using a MakerBot 3D printer. The advantage of these sensors compared to other available technologies is in its easier analysis of long-term living-cell images and quicker response in biomedical applications. Heger et al. [100] designed and manufactured 3D printed immunosensors with biocompatible PDMS chips, that can be subsequently used for magnetic isolation and quantum dots-based immune labelling of metallothionein (MT), which is used as a biomarker for various types of cancers, including spin cellular carcinoma. Polymer acrylonitrile butadiene styrene is used as a 3D printing material due to its better optical transparency, critical surface tension, stiffness and chemical resistance. Tsuda et al. [101] developed a 3D chemical fluidic system to control biological cells and liquid handling by utilising a FDM-based 3D printer since it is possible to make void channel structures directly by utilizing low-priced polymers as a printing material. This research group fabricated 3D printed sensors integrated with the cell growth chamber and a water-in-oil droplet generator. This generator, in turn, was interconnected with suitable modular devices and a valve-based flow selector, which helps observe the mixing, flow control, and monitoring of reaction progress for biological applications. 

**Figure 2:**
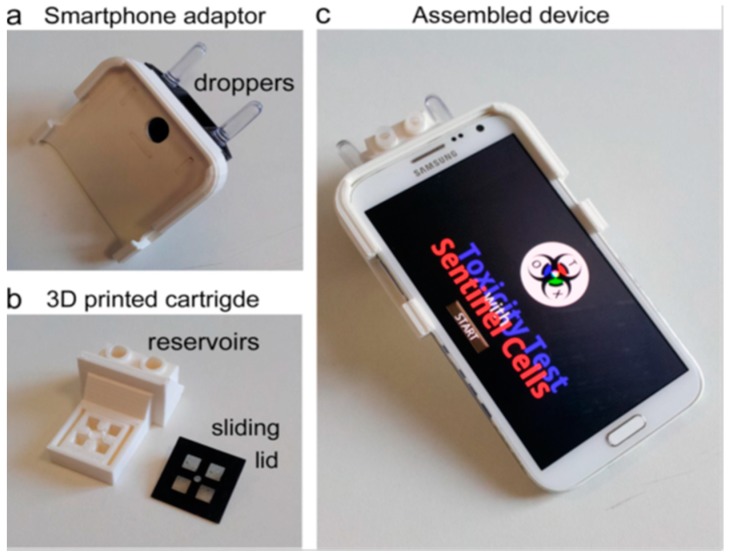
(**a**) A 3D printed smartphone adaptor depicting its (**b**) 3D printed cartridge being composed of reservoirs and sliding lid. (**c**) The assembled smartphone-based device for BL signal acquisition and analysis. Reproduced from Cevenini et al. [70].

Another interesting work explained by Cevenini et al. [70] based on a bioluminescent cell-based toxicity sensor which detects the signal by a bioanalytical application of a smartphone. This research group designed, fabricated and analysed the performance of a cell toxicity sensor embedded on a 3D printer cartridge ABS polymer. Figure 2 shows the fabrication of the bioluminescent cell-based toxicity sensor [70]. Singh et al. [102] developed a 3D printed biosensor consisting of nine layers with a central core to increase the sensitivity via increasing the enzyme-linked immunosorbent assay’s (ELISA) diagnostic performance for infectious diseases. The sensitivity detection of this 3D printed sensor increases up to 2.25 times when associated to 96-well ELISA. Roda et al. [103] fabricated a 3D printed biosensor for real-time detection of the presence of lactate in oral fluid and sweat by coupling lactate oxidase with horseradish peroxidase and observing lactic acidosis in critical-care patients. In addition to that, the sensor can be also used to avert heart attacks in critical-care patients by monitoring their lactic acidosis.

### 2.2. Stereolithography

Stereolithography is a 3D printing technique, in which a prototype or a model can be fabricated by curing the 2D layers of the polymer [104,105,106]. A STL file is sliced to obtain the information of each of the 2D layers to be printed by a focused ultraviolet (UV) laser light. This UV light supplies an external power source to initiate the photopolymerisation chain reaction. After scanning each layer, the whole built platform is required to be lowered so that the resin can fill a layer thickness above the deposited structure, which is again further laser-scanned. These steps are repeated until the entire structure is formed. The photopolymers are converted into polymer chains and get attached to the subsequent layer by radicalisation. The unreacted resin that helps to hold the structure, is removed after completion of the process. Resolution of the process is as precise as 10 μm and hence, a high-quality precise sensor can be printed by this SLA process. One of the main advantages of this process lies in the possible fabrication of large volume structures by this process.

Ragones et al. [107] developed a disposable and portable electrochemical sensor using stereo-lithography modelled in SolidWorks software for rapid detection of the biomarker alkaline phosphatase (ALP). ABS is used as a 3D printing material for bio-electrochemical sensing of enzymatic activity in tissue and cells. Dirkzwager et al. [108] developed an aptameric-tethered enzyme capture sensing system for malaria diagnosis, formed by using SLA-based 3D printing technology. Heger et al. [109] designed and fabricated carbon quantum dot (CQD)/genomic DNA complex using a 3D printed stratospheric probe. This sensitive, accurate and effective biosensor can be used to determine the level of DNA damage. Lee et al. [110] propose the 3D printing method to create an integrated microfluidic device and also proved the sensing ability of the biosensor using α-fetoprotein (AFP) biomarker detection. In this viewpoint, for bio-sensing applications in biomedical, and biochemical devices, these techniques can be used extensively to detect the AFP antigen.

**Figure 3:**
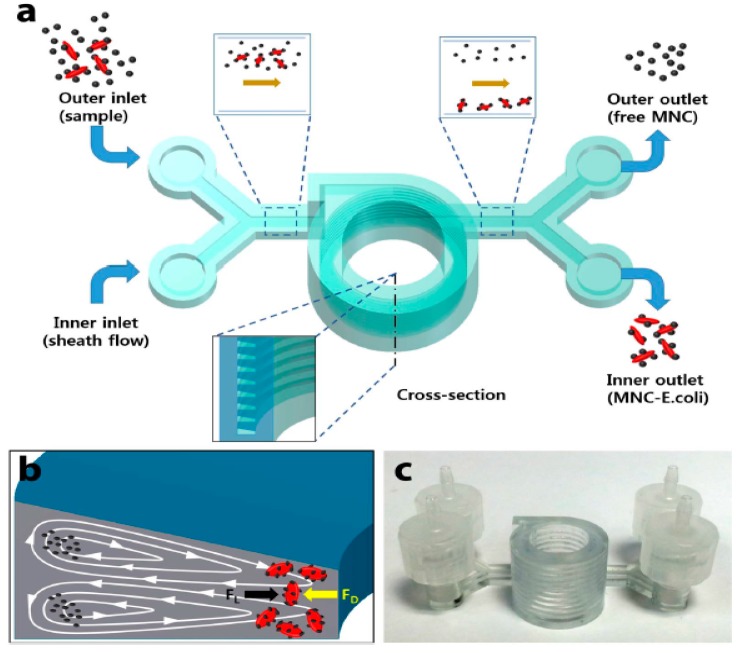
(**a**) Schematic illustration of separation of the captured bacteria by inertial focusing. (**b**) Representation of dean vortices in a channel with trapezoid cross-section. (**c**) Photograph of the 3D printed microfluidic device. Reproduced from Lee et al. [112].

Lee et al. [111] fabricated an SLA-based 3D printed microfluidic device for sensing pathogenic bacteria. WaterShed resin was used as a printing material due to its transparency and non-swelling nature when it is made in contact with an aqua solution. Due to the rapid detection capability of this 3D immune magnetic flow assay, it can be utilised in biomedical research and education. They have developed a helical microchannel device around a cylindrical chamber by using SLA-based 3D printing technology for rapid detection of *E. coli* bacteria in edible food [112]. Figure 3 shows a schematic representation of the fabrication of the 3D-printed microfluidic device [112].

Tang et al. [113] developed a fast and sensitive microfluidic device for cancer biomarker protein detection. SLA as a 3D printing method used to design and fabricate microfluidic devices due to the need for formation of a unibody design which helps maintain channel integrity and eliminates leakage. Chan et al. [114] fabricated an efficient 3D printed microfluidic component which consists of a pushing valve, rotary valve and torque-actuated pump for disposable and point-of-care of urinary protein quantification. In this way, the chips for this colourimetric analysis of urinary protein with minimal quantitative analysis becomes inexpensive. Another main advantage of using SLA is the appropriate enhancement of the optical path of the reaction chamber and volume ratio of the sample solution. Au et al. [115] manufactured a 3D printed cellular calcium image sensor-based fluidic device using WaterShed resin to record the calcium response in the green fluorescence channel. These SLA-based 3D printed PDMS fluidic valves and pumps are entirely made of plastic and efficient to valve into microchannels.

### 2.3. Polyjet Process

In this AM process, the photopolymer is used to fabricate the 3D model by a photocuring or hardening process. Instead of using one nozzle, like in the FDM process, the polyjet process uses multiple nozzles for printing. The print head moves across the x-y direction of the platform and ejects tiny droplets of photopolymer to deposit the printing material in the design based on the corresponding STL file [116,117]. After curing the deposited layer by ultraviolet lamps, the platform is then lowered, and the next layer is deposited on the previously hardened layer. The wax material, acting as a support structure, is needed to be removed after the entire 3D structure is built. Since multiple jetting heads are used for printing, this allows building multi-coloured objects in a single structure. One of the main advantages of this process is that a high resolution of 16 μm can be achieved for the prototypes, having an accuracy of less than 0.1 mm.

**Figure 4:**
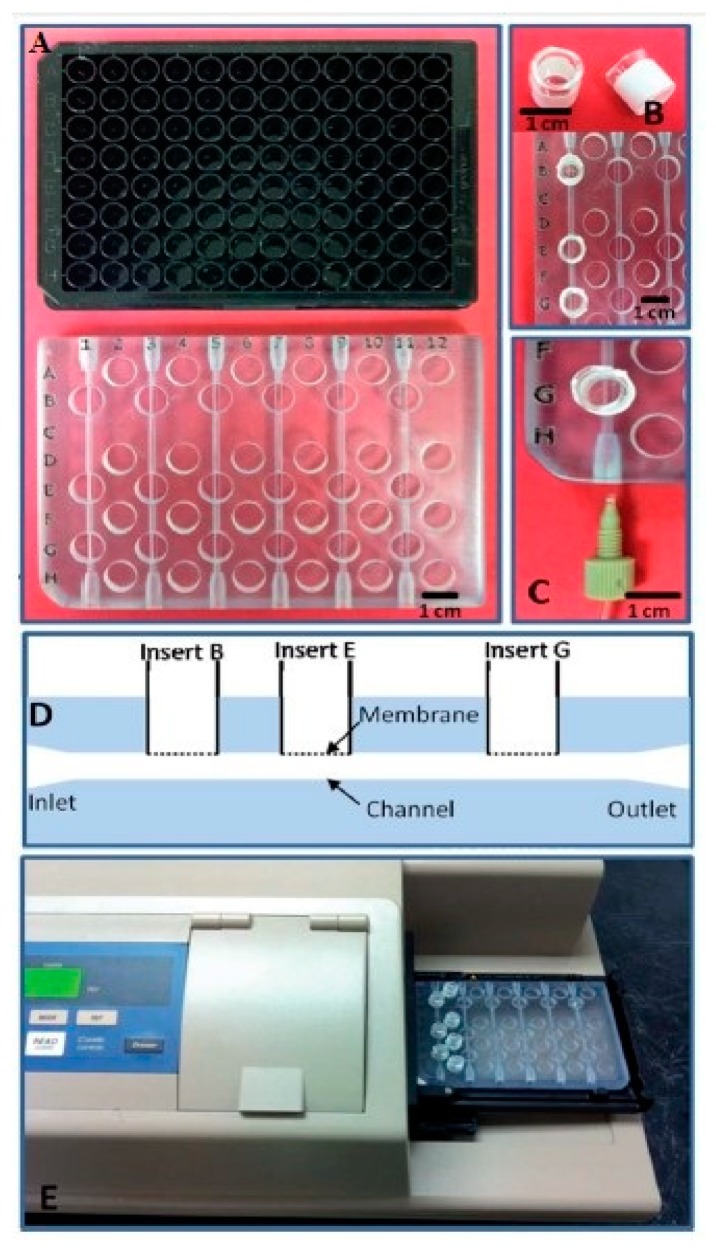
(**A**) The 3-D printed device has been modelled after the dimensions of a 96-well plate. (**B**) The inserting of the membrane is done into wells via a semi-permeable polyester membrane. (**C**) The channels are connected through threads, located at two ends of the channel. (**D**) A schematic cross-sectional view of the insertion of the channel and the membrane. (**E**) The locking of the device into the sample holder. Reproduced from Chen et al. [119].

Anderson et al. [118] fabricated a cell viability sensor-based fluidic device using a soft polymer as a 3D printed material which provides a standard fitting and ruggedness. This cell viability sensor can examine drug transport and cellular status at the same time. Chen et al. [119] developed a robust, rugged and leakage proof 3D printed storage device which is capable of several numerical determinations of adenosine triphosphate (ATP) release over a long term study for use in transfusion medicine. This cell-based ATP sensor works using chemiluminescence as a transduction mechanism to determine the ERY-derived ATP in a blood component using a solution containing luciferin–luciferase. Figure 4 represents the explained 3-D printed fluidic device [119]. 

Erkal et al. [120] developed two 3D printed devices using a proprietary acrylate-based polymer material for analysing ATP and dopamine sensing. Oxygen recognition in a streamline of hypoxic red blood cells and concentration detection of nitric oxide (NO) using the reusable electrodes has a great advantage in biomedical applications. Salvo et al. [121] designed and fabricated a dry electrode-based physiological sensor by UV cured layers using insulating acrylic-based photopolymer since it provides low priced and highly precise 3D shapes. Due to its capability for recording electrocardiogram-electroencephalogram (ECG-EEG), this prototype can be an advantageous model for medical purposes. An electrochemical and biocompatible 3D sensor was developed by Ragones et al. [122] using PDMS substrate filled with a proprietary conductive polymer to detect alkaline phosphatase (ALP) enzyme levels activity of different cancerous cells like a diagnostic tool. This 3D printed chip holder allows real-time detection and non-invasive measurement in healthcare applications by facilitating an accurate surgical procedure. 

### 2.4. Selective Laser Sintering

Selective laser sintering is an additive manufacturing process in which three-dimensional structures are formed layer by layer by bonding between powder particles within the same layer and simultaneously with the consequent layer [123,124,125,126]. A certain laser power is required to melt the periphery of the particles using the localised energy of a laser beam. The unused powder acts as a support structure for the 3D printed part. After scanning each layer, the structure is lowered to spread a new powder layer which can be scanned according to the computer-aided design (CAD) design. Not only metallic powder particles but also ceramics and polymers or combinations with each other can be used in SLS. The hatching gap, which is the distance between two subsequent scanning paths and the layer thickness is the important parameter to control the material properties of the resulting 3D structure. Most of the SLS process is carried out in an inert atmosphere to avoid oxidation processes caused by environmental oxygen. One of the great advantages is that a wide range of materials can be fabricated precisely where the unused powders can be recycled in this 3D printing technique.

**Figure 5:**
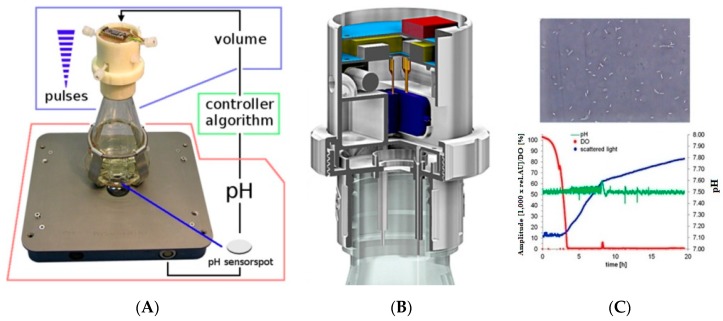
Continuous recalibration of the 3D-printed Control Unit Adaptive P controller. Reproduced from Ude et al. [127]. (**A**) The 3D printed flask is used to control the pH of the solution using defined algorithm. (**B**) The interior of th3 3D printed flask. (**C**) Variation in the amplitude, pH levels and intensity of the scattered light with time.

Ude et al. [127] used polyamide (PA12) to fabricate a shake flask control (SFC) using the SLS technique as a 3D printing process to monitor pH online for liquid dispensing instruments. The application of this cell density sensor can be extended to control cell disruption methods, exactly distribute different chemicals and for controlling enzymatic assays. Figure 5 shows the continuous recalibration of the 3D-printed control unit adaptive P controller [127].

### 2.5. 3D Inkjet Printing

In 3D inkjet printing, powder particles needed to be spread on the platform and a low viscosity photocurable resin or hydrogel droplets are used as the printing material. This liquid composition helps to binds the powder particles to form a solid structure of sufficient strength [128]. Each layer can be built by ejection of the ink from a fine deposition nozzle, and the 3D model can be fabricated in a layer by layer approach. There are two types of inks used in inkjet printing, one is liquid-based, and another is wax-based. In liquid-based inks, the liquid binder gets evaporated and solidifies the structure, whereas in the wax-based ones, the wax needs to be melted in a heated nozzle and selectively deposited. Adhesion between he binder and the powder particles is an important factor for maintaining the quality of this layered manufacturing process. 

**Figure 6:**
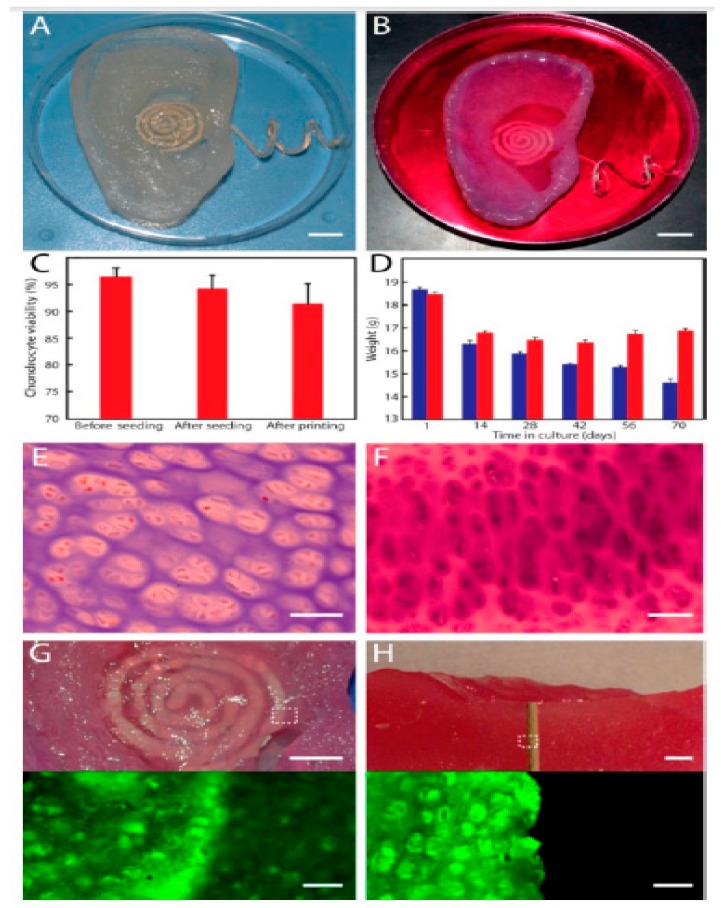
Image of the (**A**) fabricated 3D printed bionic ear and (**B**) 3D printed bionic ear during its vitro culture. (**C**) The viability of chondrocyte at different stages during the printing process. (**D**) Deviation of the weight of the printed ear over time in culture, where the ear consisted of the chondrocyte-seeded alginate or only alginate shown in red and blue colour respectively. (**E**) Histologic analysis of chondrocyte morphology done using H&E staining. (**F**) Neocartilaginous tissue being Safranin O stained after 10 weeks of culture. Photographs (top) and fluorescent (bottom) images of (**G**) viability of the neo cartilaginous tissue being in contact with the antenna of the coil and (**H**) cross-section of the bionic ear showing the viability of the internal cartilaginous tissue in contact with the electrode. Reproduced from Mannoor et al. [82].

Mannoor et al. [82] printed a 3D bionic ear as shown in Figure 6 [82], using a cell-seeded alginate hydrogel-based printing material and biological cells as inks to improve the hearing sensing for radio frequency reception along with stereo audio music. An electrically conductive silver nanoparticle (Ag NP)-infused inductive coil antenna is attached to the printed bio-electronic hybrid ear to catch the electromagnetic signals of a certain range. Xu et al. [129] fabricated a sensor and actuator integrated heart structure-shaped 3D elastic multifunctional biomembrane to receive information regarding spatial and temporal responses like thermal metabolic changes caused by therapies and diseases. 

Dankoco et al. [130] used the inkjet printing process to print a flexible temperature sensor to measure the human body temperature within a range of 20 to 60 ℃. The leading advantage in the medical research and application is that it requires the lowest possible current under a bias voltage of 1 V with good linearity and a hysteresis of less than 5%. Briand et al. [131] developed a flexible resistive temperature sensor having a good linearity in the range of −10 to 140 ℃, using silver inkjet printing in combination with thick Ni electroplating on PET foils. Li et al. [132] established a 3D printed stretchable capacitive sensor for highly sensitive tactile and electrochemical sensing applications. The interdigital strain sensor has great stability since during the measurements the capacitance is very steady with little deviation.

### 2.6. Digital Light Processing

Digital light processing is similar to SLA except for the different photocuring process. Here, a digital projector screen flashes to project a layer, made of squared voxels, like an image [133,134]. Each 2D hardened layer is formed after exposing the liquid polymer to projector light under the safest conditions instead of making a layer with several laser scan paths. The process is repeated until the entire structure is fabricated. 

**Figure 7:**
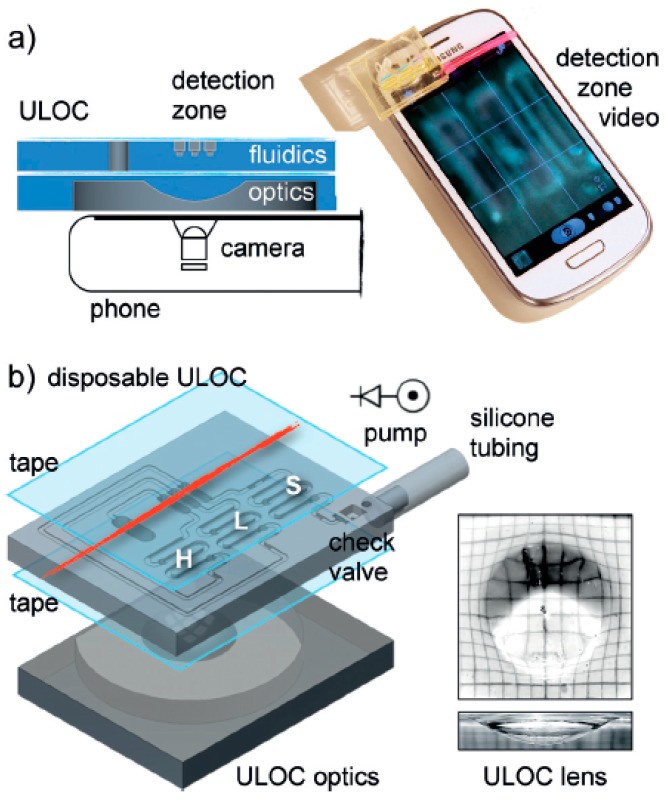
Schematic diagram of the (**a**) 3D printed optics for universal coupling to cell phone camera and (**b**) 3D scheme of the unibody lab-on-a-chip (ULOC) device detailing the fluidic component. Reproduced from Comina et al. [135].

Cominaet al. [135] developed a prototype optical components-based glucose sensor by curing a photoresin using a colourimetric transduction mechanism as shown in Figure 7 [135]. This highly beneficial glucose biosensor has great advantages in medical and clinical applications. Dantism et al. [136] fabricated a 3D printed light-addressable potentiometric sensor (LAPS) system by the layer by layer technique using a photopolymer (PP-ABS). The system helps measure the cell number at a particular glucose concentration as well as changes in the concentration of glucose at a fixed cell number by quantifying the metabolic activity of *E. coli* K12 bacteria. To measure the cell growth, Takenaga et al. [137] developed a semiconductor-based biosensor by the 3D printing method using photoresin consisting of polypropylene/acrylonitrile butadiene styrol as a printed material by exposing it to a UV light pattern.

Tiller et al. [84] fabricated a piezoelectric acoustic sensor-based microphone from 3D-printable composite resins using the DLP method which can send electric signals. Building a single layer membrane was the challenge for this research group since the threshold of dose energy varies in the different polymers which decides the thickness of the polymer in DLP. They noted that for printed membranes, thicknesses down to 35 µm are sensitive enough to work as an electro-acoustic device. Also, this device can be embedded into a pre-amplifier printed circuit board to work as a microphone in a bio-inspired design.

Liu et al. [138] developed a 3D printed force sensor from transparent high-temperature resin using the DLP process due its cost-effectiveness, precision and fast production. To investigate the structure of the sensor, FEM models were developed, and for the optimisation of the process parameters, the resistance of the sensor was analysed. The printed sensor shows a sensitivity of 2.92% N − 1 with a linearity error of 3.1485% full scale. Thirty-five specimens (60 mm × 15 mm × 3 mm) were developed by varying the curing time in the range of 0 to 3 h to measure the individual elastic modulus and flexural strength. Utilising them in robot whiskers is one of the major applications of these force sensors.

Ge et al. [85] fabricated a soft pneumatic actuator based on the DLP technique that can develop enough deformation under high pressure. This micro soft pneumatic gripper was tested to verify the grasping capability of the 3D printed gripper. The dimensions of the gripper that can be printed within 30 min are 0.4 mm × 0.4 mm. Due to its flexibility, it can be used in invasive surgery applications. The FEM analysis shows that this 3D printed actuator shows a significant deformation under relatively low pressures, which is why it has good grasping capability. 

## 3. Current Challenges and Future Opportunities

Even though a lot of work has been done regarding 3D printed sensors for biomedical applications, there are still some gaps that need to be filled in the current scenario. One of the primary limitations is related to the biocompatibility of the printed materials. Even though the printers mostly rely on commonly used printing filaments, the utilisation of the printed sensors as implantable prototypes remains an issue [139]. The dependence of the fabrication of sensors based on plastics is another issue which is dangerous to living beings and environment. Even after the use of the sensors, their recyclability and reusability remain an issue. The sensors that are developed to be recyclable needs to be constantly tested and verified for determining their efficiencies and sensitivities in the specific applications. Saturation of the responses of the sensors occurs when they are used for a long time. One of the techniques to deal with this problem is the association of dynamic thresholding [140] with the responses of the sensors. Two specific situations corresponding to the outputs of the sensors can be classified by maintaining a certain threshold level. Another limitation related to the 3D printing technique is the emission of harmful and carcinogenic nanoparticles which can cause disastrous effects in a human body [141]. Avoiding this problem can only be dealt with by developing printing machines which work with biocompatible materials while producing minimum emissions of particles. Some recently developed printers use polymers and wax materials to print prototypes. Even though there are additional post-processing steps attached to these devices, the emissions of harmful nanoparticles are greatly reduced with these devices. Another disadvantage related to most types of 3D printing is the initial high cost of production of the devices. FDM is a technique which faces the highest cost of production since only a limited number of ceramics and plastics can be processed with this technique. The fabrication of sensors using FDM can only be a cost-effective process if the sensors are produced on a large scale in the industrial sector. Some of the limitations of the SLS process are related to the porous nature of the developed sensors, which can cause constraints in some of the specific applications related to electrical sensing. This can be dealt with by placing an additional layer over the fabricated sensor, which can be made selective as well to obtained optimised electrical, mechanical and thermal characteristics. Another disadvantage of SLS is the thermal distortion caused by some of the processing materials due to the heat generated [142]. This affects the size of the developed sensors, which eventually affects their performance. One of the ways to deal with this problem is the association of some of the heat-resistant materials along with the processed polymers. Some polymers like PI can be used as an additive layer with the raw materials to impart thermal resistance to the developed sensors. Some of the limitations related to stereolithography are the time consumption to fabricate each sensor, expensive equipment and fragility of the developed sensors [143]. The problem related to the time consumption can be dealt with by the mass production of sensors, which can reduce the per unit time required to develop each sensor. The fragility of the sensors can be dealt in the same way as SLS, where other processing materials can be associated with the fragile materials to form a stronger finish product. Polymers like PDMS offer a favourable choice due to its malleable nature.

Similarly, graphene [144] and CNTs [145] are some of the conductive materials that can be conjugated with the developed sensors to make them electrically conductive. Another major disadvantage of stereolithography is the surface roughness, which results in its fragility. This problem is also associated with the 3D polyjet printing process where the supporting materials cause alterations of the surface and the subsequent properties of the resulting sensor. One of the techniques to address this problem is to create multi-layered structures which even though they would require the conjugation of a few printing materials, they would address the surface problems caused by the characteristics of the materials with such limitations. These problems, once addressed, can enhance the utilization of 3D printing techniques to develop sensors for biomedical applications to a large extent. 

One of the brighter prospects for the future has been the bloom of 4D printing [146,147,148], which is technically an enhanced form of additive manufacturing of different materials. Researchers have been working on 4D printing [149] where certain materials like shape memory polymers have been processed to create active heterogeneous structures. These structures are being developed in a controlled manner, whose configuration changes accordingly with the environmental stimulus. The usage of 4D printing is advantageous as the fabricated structural configuration can be changed simultaneously with the considered application. The structures developed can be used to form specific patterns like origami components, where a printed elastomeric matrix containing active composites and polymer fibres changes its shape. The biggest advantage in 4D printing is the allowance of structure shape customisation at any point in time, which allows the interchanging of the shapes from one dimension to three dimensions and vice versa at any point. Some 4D printing techniques emphasise on DLP and polyjet printing due to their advantages for processing a wide range of materials to form different kinds of rigid, soft, transparent and high-resolution structures [150]. Another advantage of 4D printing technique that is fused with 3D printing is the resilience of the developed structures, which would help in the biomedical sector to a great extent. The constant changes in the human body conditions, economic and environmental factors demand a subsequent similar change in sensors in terms of their robustness, sensitivity and morphological features. One of the works explaining the conjugation of 3D and 4D printing techniques for biosensing purposes has been explained by Mandon et al. [151], where biomimetic applications have been carried out using 3D-printed enzymes. A DLP printing technique was used where the ink was formed by mixing aqueous solutions of cresol red and Irgacure with glucose oxidase, peroxidase and glucose oxidase at specific concentrations. The advantage of this technique for 4D printing lies in the customisation of the image, layer attached and perimeter exposure times. Once the CAD was uploaded to the device working with visible light irradiation, multiple components were printed with the help of phosphate-buffered saline (PBS) buffer. The prototypes were then processed with a chemiluminescent assay and calcification process to form 3D architectures and possibilities for tissue engineering and bone reconstruction. This work shows that 4D printing increases the dynamicity of the developed structures, thus providing an additional step to the ones described in the preceding section. The future of 3D printing technology for sensors is very bright in terms of its market size for the next ten years. It has been predicted that there will be a substantial rise in the GDP for 3D printing from 137.1 million USD in 2017 to 3915 million USD in the next ten years [152]. This includes a lot of work in the development of certain sensor and sensing prototypes like printed circuit boards, semiconducting and OLED screens. Among the 3D printing techniques currently available, FDM has its highest number of users [153]. The reason for the highest use of FDM can be attributed to the easy availability of plastics, that are processed to fabricate the prototypes in this process. It has been predicted that around 200,000 parts are 3D printed every quarter of a year using one of these techniques. It is likely that this number will increase, given the substantial advantages of this technique. In terms of usage of materials, a range of materials are nowadays considered for developing 3D printed sensors [154]. Among them, maximum proportions are being used by some polymers like PET and PI, along with ceramics and thermoplastics. The usage of other materials should be increased to develop nanocomposite-based 3D printed sensors with optimized characteristics. 

## 4. Conclusions

The paper presents a substantial review of some of the 3D printed sensors for biomedical applications. The 3D printing techniques have been classified into six types depending on the way the materials are processed to form the final prototypes. The different types of 3D printing methods involved in developing sensors are fused deposition modelling, stereolithography, selective laser sintering, polyjet processes, inkjet printing and digital light processing. Each of these processes has its own merits and demerits related to cost and time of fabrication, the type of materials that can be processed and prototypes that can be formed. A few of the current bottlenecks have also been mentioned, along with the possible remedial solutions to deal with them. Finally, a market survey has been presented about the expenditures on the different types of 3D printing techniques in the current scenario and in the upcoming years to develop sensors and other electronic appliances.

## Figures and Tables

**Table 1:** A summary of additive manufacturing techniques: principle, materials, resolution and 3D printed sensors in biomedical applications.

3D Printing Methods	Principle	Materials	Resolution Range (μm)	Application of 3D Printed Sensor in Biomedical
Fused deposition modelling	Extrusion of constant filament	ABS, PLA, Wax blend, Nylon	x: 100 y: 100 z: 250	Lactate sensor [69], Cell toxicity sensor [70], Immunosensor [71], DNA sensor [72], Glucose sensor [73], Bacteria sensor [74]
Stereolithography	UV initiated polymerisation cross section by cross section	Resin (Acrylate or Epoxy based with proprietary photoinitiator)	x: 10 y: 10 z: 15	DNA imaging sensor [75], Bacteria sensor [76], Cellular sensor [77]
Polyjet	Deposition of the droplets of the photo-curable liquid material and cured	Polymer	x: 30 y: 30 z: 20	Cell imaging sensor [78], Cell based sensor (for ATP sensing) [79], Physiological Sensor [80], Immunosensor [71]
Selective laser sintering	Laser-induced sintering of powder particles	Metallic powder, polyamide, PVC	x: 50 y: 50 z: 200	Cell density sensor [81]
3D Inkjet printing	Extrusion of ink and powder liquid binding	Photo-resin or hydrogel	x: 10 y: 10 z: 50	Bionic ear [82], Multifunctional bio-membrane [83]
Digital light processing	Photo-curing by a digital projector screen to project layers by squared voxels	Photopolymer and photo-resin	x: 25 y: 25 z: 20	Piezoelectric acoustic sensor [84], motion control and soft sensors [85], Glucose sensor [86]

**Table 2:** Comparative study of the 3D printing methods in terms of their advantages, disadvantages, accuracy and repeatability.

3D Printing Methods	Advantages	Disadvantages	Accuracy (µm)	Repeatability
Fused deposition modelling	High speed High quality Used for a wide range of material Durable over time Less time	Porous structure for the binder Weak mechanical properties Often required support	350	Fair
Stereolithography	Large parts can be built easily High accuracy and surface finish Good for complex built Simple scalability Uncured material can be reused	Not well-defined mechanical properties due to the usage of photopolymers Slow build process Expensive process Moisture, heat, and chemicals can reduce its durability Brittle structure	25–150	Good
Polyjet	Multiple jetting heads are available to build materials Different levels of flexibility Allows using different coloured photopolymers More control over the accuracy High accuracy and smooth surface	Vulnerable to heat and humidity Lose strength over time Relatively higher cost compared to others Sharp edges are often slightly rounded.	10–20	Good
Selective laser sintering	High resolution No support structure is required High strength Less time Complex structures can be easily fabricated	Only metal parts can be printed Finishing or post-processing required due to its grainy roughness Difficulty in the material changeover.	300	Good
3D Inkjet printing	Very good accuracy Very high surface finishes.	Fragile parts Slow build process The grainy or rough appearance Post-processing is required to remove moisture Poor mechanical the properties.	100	Excellent
Digital light processing	Excellent accuracy of laying High resolution Uncured photopolymer can be reused.	Insecurity of the consumable material Difficult to print large structure Boxy surface finish due to its rectangular voxels.	10–25	Excellent
